# An Organic Chemist's Guide to Mediated Laccase Oxidation

**DOI:** 10.1002/cbic.202200411

**Published:** 2022-10-18

**Authors:** Katharina Obleser, Hubert Kalaus, Bernhard Seidl, Martin Kozich, Christian Stanetty, Marko D. Mihovilovic

**Affiliations:** ^1^ Institute of Applied Synthetic Chemistry TU Wien Getreidemarkt 9 1060 Vienna Austria; ^2^ Agrana Research & Innovation Center GmbH Josef-Reither-Straße 21–23 3430 Tulln Austria

**Keywords:** AZADO, laccase-oxidation, mediators, TEMPO, *T. versicolor*

## Abstract

Laccases are oxidases that only require O_2_ as a terminal oxidant. Thus, they provide an attractive green alternative to established alcohol oxidation protocols. However, laccases typically require catalytic amounts of mediator molecules to serve as electron shuttles between the enzyme and desired substrate. Consequently, laccase‐mediator systems are defined by a multitude of parameters such as, e. g., the choice of laccase and mediator, the respective concentrations, pH, and the oxygen source. This complexity and a perceived lack of comparable data throughout literature represent an entry burden into this field. To provide a solid starting point, particularly for organic chemists, we herein provide a time‐resolved, quantitative laccase and mediator screening based on the oxidation of anis alcohol as model reaction. We measured the redox potentials of mediators under the reaction conditions to relate them to their performance. Lastly, for particularly efficient laccase‐mediator pairs, we screened important reaction parameters, resulting in an optimized setup for mediator‐assisted laccase catalyzed oxidations.

## Graphical abstract

**The mediator‐assisted** laccase‐catalyzed oxidation of alcohols is a green alternative to established classical synthetic protocols. However, the interaction between laccases, mediators and oxygen comprises a highly complicated, multivariate system. We present a detailed comparative study evaluating the most important components and parameters via an anis alcohol model system.

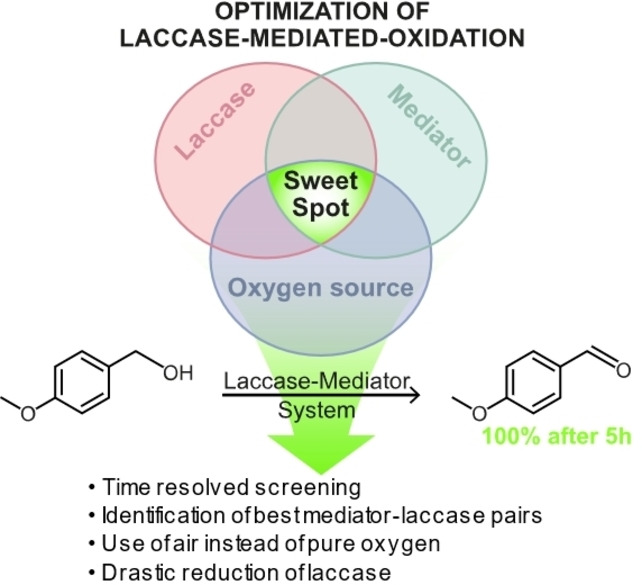

## Introduction

Laccases belong to the superfamily of blue‐copper oxidases (EC 1.10.3.2, *p*‐diphenol: dioxygen oxidoreductases).^[1]^ They were first discovered in 1883 by Yoshida *et al*. in the resin of the lacquer tree *Rhus vernicifera*.^[2]^ Today, laccases are commonly found in fungi, higher plants, bacteria, and insects. Laccases are redox enzymes that can oxidize substrates with O_2,_ serving as a stoichiometric oxidant that is reduced to water.^[3]^ This oxidation proceeds *via* a cluster of copper(II)‐atoms situated in the active site.^[4]^ The oxidized substrate, usually a radical species derived from a 1,2‐disubstituted aromatic compound or from cinnamyl alcohol, then undergoes various reactions: e. g., forming dimers or polymers or triggering intra‐molecular rearrangements.^[4b]^ So far, a diverse range of reactions directly catalyzed by laccases have been identified, which opened up new synthetic pathways towards, e. g., heterocyclic cores, polyphenols including several bioactive compounds.^[5]^ These potential reactions should be considered as a possible side reactions when mediator‐assisted oxidation is intended.

**Scheme 1:**
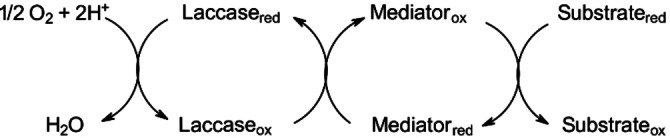
A schematic representation of the catalytic cycle of the laccase‐mediated oxidation reaction.

Initially, the use of laccases was restricted to this limited range of (natural), easy‐to‐oxidize substrates, which is due to ′laccases′ relatively low redox potentials (350–800 mV *vs*. normal hydrogen electrode (NHE)). As an example, phenols have a low redox potential and are small enough to fit in the enzyme‘s active site, thus can be very readily oxidized, a requirement that is only fulfilled by a rather narrow group of substrates.^[6]^ The solution for widening the substrate range was the introduction of mediator molecules.^[7]^ These small organic molecules can be readily oxidized and reduced and act as electron shuttle from the laccase to other substrates (Scheme 1). This way, unprecedented transformations *via* laccases could be developed, the oxidation of primary aliphatic alcohols to aldehydes being a particularly important application. Today, the excitingly broad palette of laccase‐mediator applications reaches from the degradation of waste,^[8]^ plastics,^[9]^ polyaromatic hydrocarbons,^[10]^ dye and colorant bleaching^[11]^ to the production of new composites by functional biotransformations.^[12]^ Laccase‐mediator systems even find application in the regeneration of cofactors in coupled, multi‐enzymatic redox transformations.^[13]^

In the light of an ever‐growing demand for more sustainable products and processes, laccases are an enzyme class of particular interest for both industry and the scientific community. Advantages of the use of laccases are the low toxicity of reagents, the application of water as a solvent, and the atom efficiency because of the catalytical nature of enzymes and the mediator. This allows the replacement of potentially toxic, mostly unrenewable chemical oxidants and organic solvents. Within biocatalysis, laccases stand out, requiring only oxygen as terminal oxidant, rendering their application cheaper and operationally simpler to perform than of, e. g., peroxidases requiring cofactors for their regeneration.^[14]^ Therefore, the application of laccases opens new possibilities to reduce the environmental impact while at the same time evading costly cofactors both on a laboratory scale as well as in industrial processes.

To date, different types of mediator molecules from different structural families bearing various functional groups are known (Figure 3). Different mediator families exhibit various mechanisms in the laccase‐mediated alcohol oxidation, widening the scope of laccase catalyzed oxidation to mechanisms not available to the laccase alone.^[6]^ Generally, three different oxidation mechanisms for mediators are known. While 2,2′‐azino‐bis(3‐ethylbenzothiazoline‐6‐sulfonic acid) (ABTS, **11**), one of the first mediators identified, is suggested to oxidize the alcohols over an electron‐transfer mechanism (ET),^[6]^ for the *N*‐hydroxy compounds 1‐hydroxy benzotriazole (HBT, **12**), *N*‐hydroxyphthalimide (NHP,**10**)^[15]^ and violuric acid (VLA, **13**) a hydrogen abstraction pathway (HAT) was suggested.^[6]^

**Scheme 2:**
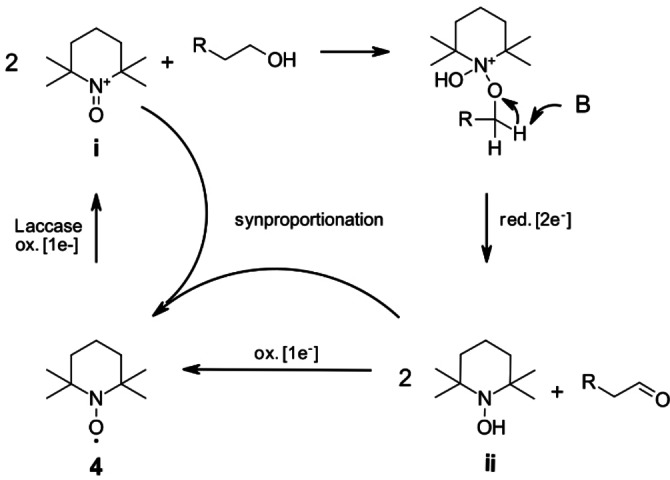
Ionic mechanism of the catalytic cycle of the mediator 2,2,6,6‐tetramethyl piperidine‐1‐yl)oxyl (TEMPO, **4**)^[16a]^ with indicated reduction/oxidations steps referring to the mediator.

The most prominent mediator class are stable *N*‐oxyl radicals, such as 2,2,6,6‐tetramethylpiperidin‐1‐yl)oxyl (TEMPO, **4**) and 2‐azaadamantane *N*‐oxyl, which despite their radical nature follow an ionic mechanism,^[16]^ as depicted for TEMPO in Scheme 2.^[17]^ The radical species is oxidized by laccase to the oxoammonium ion (**i**) as an active oxidizing agent, reacting with the alcohol and resulting in the formation of hydroxylamine (**ii**) and aldehyde. This step was identified as the rate determining step by Tromp *et al*. in 2010. The catalyst recycling is mainly achieved *via* synproportionation of hydroxyl amine (**ii**) and oxoammonium (**i**) to *N*‐oxyl radical (**ii**), which is then re‐oxidized to the ionic oxoammonium relevant for the substrate oxidation.^[16a]^ Radical species are generally considered highly reactive and unselective in their reactivity, despite notable exceptions to this.^[18]^ In contrast, *N*‐oxyl radicals are very stable,^[19]^ exhibiting only very few defined radical‐reactivities, as in the controlled nitroxide‐mediated radical polymerization of alkenes, with substrates particularly prone to form radical species or other radicals when used as a radical scavenger in methodological studies.^[20]^

The obvious strengths of mediated laccase‐oxidations have been presented on specific combination of reagents in a variety of original reports and selected reviews covering oxidation of a great variety of primary alcohols, including from unprotected carbohydrates, secondary benzylic alcohols and for particularly reactive mediators like AZADOL also selected secondary alcohols.[^1^, ^4a^, ^6^, ^16b^, ^21^]

**Figure 1:**
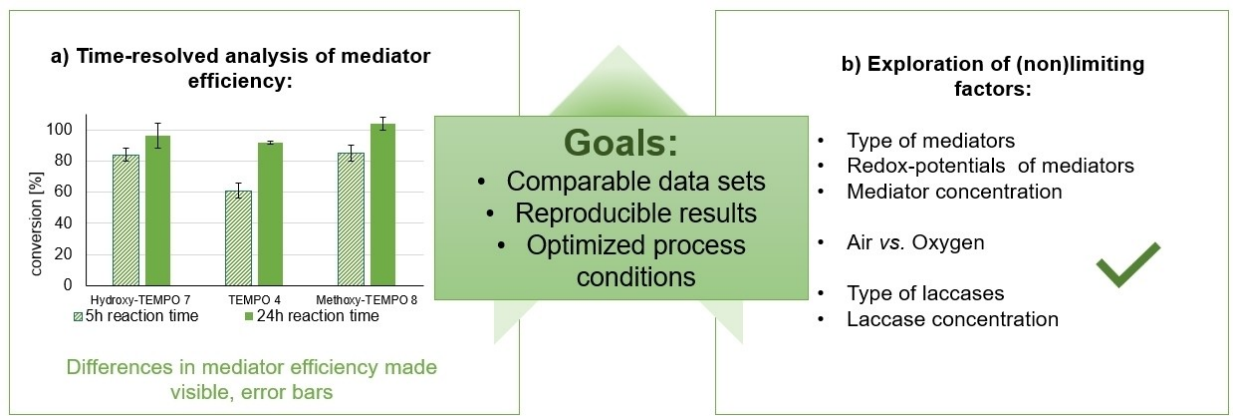
The vision of this work.

Independent of the substrate, type, and concentration of studied substrate, as well as mediator, type, and activity of provided laccase at different optimal pH are just the main parameters that differ throughout literature, hampering direct comparison of reported results. Further, within many reports only single‐time (end)points of conversion are reported, which we deem insufficient to accurately sort mediators according to their performance (Figure 1).

Therefore, even despite the extensive efforts directed towards developing powerful laccase‐mediator systems,[^1^, ^22^] we discovered a lack of a systematic, time‐resolved comparison of commercial mediators and experimental conditions, also in the light of the limiting and possibly non‐limiting factors. Consequently, the large number of parameters that have to be analyzed/understood represent a high entry burden for an organic chemist against venturing into this field.

Therefore, we set out to provide a comprehensive comparison of the efficiency of commercial mediator molecules as well as laccases under standardized conditions in a time‐resolved manner (Figure 1). Based upon these results, we have optimized the conditions for the best mediators and laccase pairs in respect to concentration and evaluated the effect of the oxygen source.

Consequently, we are convinced that this paper will give valuable and streamlined guidelines for applying laccase‐mediated oxidation and *en route* deciphered some of the complexity of the overall setup and generate robust comparability in this field.

## Results and Discussion

The first step was to establish the setup for a fast and reliable screening system based on a suitable model substrate. A good model substrate must allow fast, reliable, and reproducible analysis. It should be reactive but not prone to recovery losses, neither due to its physical properties of starting material or products nor due to follow‐up reactions like the overoxidation to the corresponding acid.

In this light, anis alcohol proved an ideal model substrate for our purpose: This substrate had already been used as a substrate for laccase‐mediated oxidations,^[21b]^ it allowed straightforward quantitative reaction monitoring based on GC‐FID analysis without significant recovery losses of alcohol or aldehyde and showed no overoxidation to the corresponding acid. On the contrary, benzyl alcohol, another precedented model compound, was not a suitable substrate for us due to reproducibly low recoveries of benzaldehyde (also in control experiments) which is in consistence with previous reports.^[16a]^

**Scheme 3:**
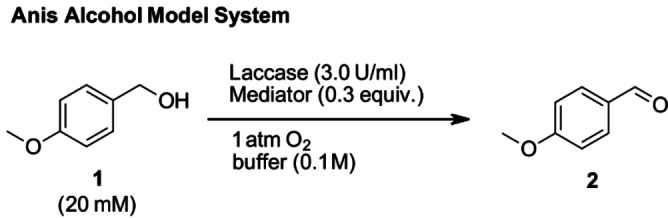
Model system set‐up: Reaction conditions for the laccase‐mediated oxidation of anis alcohol **1** to anisaldehyde **2**.

We decided to start with conditions analogous to the study of Fabbrini *et al*.: The reaction was performed at 20 mM substrate concentration, with 0.30 equiv. of mediator, 3.0 U/mL laccase activity and 1 atm. O_2_.^[21b]^ The principle set‐up of our model system using anis alcohol (**1**) as the model substrate is depicted in Scheme 3 and all reported results were obtained in triplicates.

### Laccase screening

**Figure 2:**
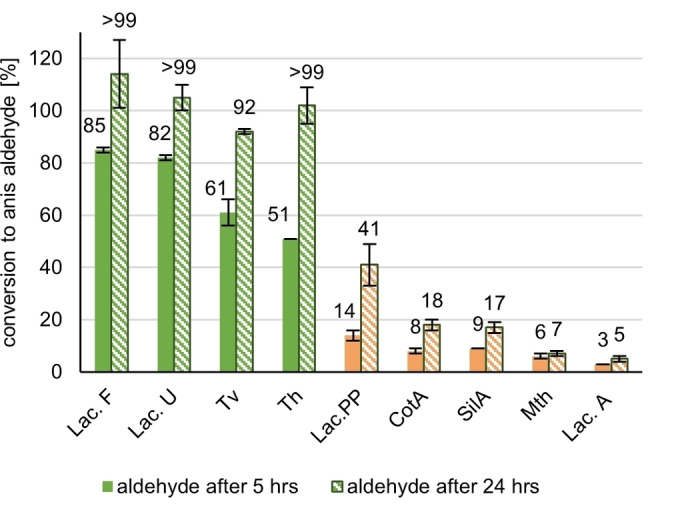
Screening of laccases; reaction conditions: [Anis alcohol]=20 mM, [TEMPO]=6 mM (0.3 equiv.), [Laccase]=3 U/mL, 1 atm O_2_, rt, reaction time: 24 h, reaction monitoring via GC‐FID (internal standard method: methyl benzoate) after 5 and 24 h reaction time, All reactions were made in triplicates with standard deviation being indicated. Resulting mean values slightly exceeding 100 % were labeled as >99. Laccases: *Trametes versicolor* (*Tv)*, *Trametes hirsuta (Th)*, Bacillus Spore coat Laccase *(CotA)*, *Streptomyces ipomoeae* (SilA), *Myceliphtora thermophilia (Mth)*, Laccase U*, Laccase A*, Laccase F*, Laccase PP*, * from ASA Spezialenzyme.

First, we set out to determine the influence of the choice of laccase on the conversion of anis alcohol (**1**) to anisaldehyde (**2)** by screening nine different laccases with TEMPO as the most prominent mediator (Table 1). Their enzyme activities were determined photometrically at 25 °C using ABTS as substrate at their respective pH optimum (range of pH: 3.5–7.0) in the required specific buffer. The redox potential is the relative number for the oxidative potency of a laccase which, if reported by the supplier, are listed together with the optimal conditions in Table 1. In Figure 2, the results of the laccase screening using TEMPO (**4**) as mediator are compiled. The four best performing laccases are Laccase F, Laccase U from ASA‐Spezialenzyme and *Trametes versicolor (Tv)* from Sigma Aldrich and *Trametes hirsuta (ThL)*, which we received from the Gübitz group of the university of Natural Resources and Life Sciences, Vienna. All four achieved complete conversion of alcohol (**1**) after 24 h, exhibiting slight differences in recovery of aldehyde (**2**). However, differences in conversion could be established at the additional 5 h time point. Interestingly, there seems to be a sharp cut‐off in reactivity between laccases with high redox‐potentials and those with low ones ≤460 mV. Although for the Laccases from ASA‐Spezialenzyme we do not know origin and redox potential, also those seem to match the clusters in respect to the optimal pH values reported. Due to the available full characterization and its availability from routine vendors, laccase from *Trametes versicolor* was selected from the four high‐performing laccases for our further work.

**Table 1:** Laccases used for screening, buffers used in respect of their pH optima, and the redox potentials in mV, sorted by their performance in the laccase screening.

Entry	Laccase	Buffer (pH‐ optimum)	Redox potential [mV]
1	Laccase F^[a]^	NaOAc buffer, 0.1 M (pH 4.5)	^[b]^
2	Laccase U^[a]^	NaOAc‐buffer, 0.1 M (pH 5)	^[b]^
3	*Trametes versicolor (Tv)*	NaOAc buffer, 0.1 M (pH 4.5)	785^[7b]^
4	*Trametes hirsuta (ThL)*	Na‐succinate buffer, 0.1 M (pH 3.5)	780^[23]^
5	Laccase PP^[a]^	K_2_HPO_4_ buffer 0.1 M (pH 7)	^[b]^
6	*Bacillus spore coat laccase (CotA)*	K_2_HPO_4_ buffer, 0.1 M (pH 7)	455^[24]^
7	*Streptomyces ipomoea (SilA.)*	K_2_HPO_4_ buffer, 0.1 M (pH 7)	337^[25]^
8	*Myceliphtora thermophilia (Mth)*	K_2_HPO_4_ buffer, 0.1 M (pH 7)	460^[26]^
9	Laccase A^[a]^	NaOAc‐buffer, 0.1 M (pH 6)	^[b]^

[a] Unknown organism, gifted from ASA Spezialenzyme. [b] Source not disclosed by producer.

### Mediator screening

**Figure 3:**
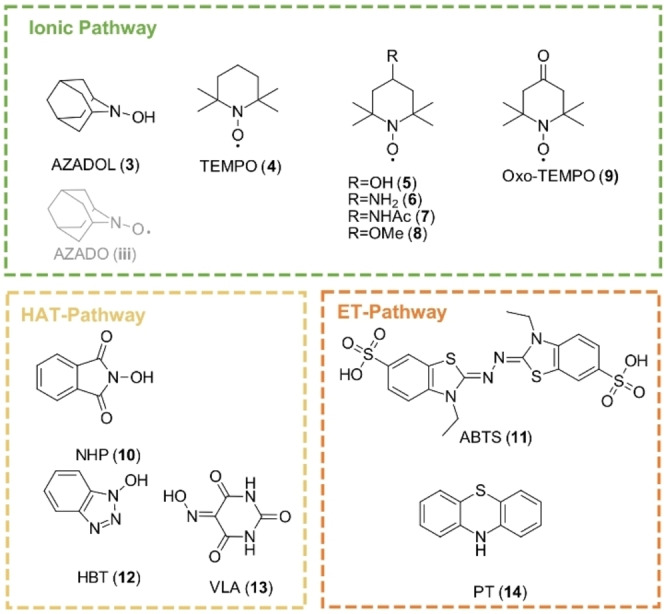
Mediators used in the time‐resolved mediator screening sorted according to the mechanisms they follow.

Next, with the laccase from *T. versicolor*, a time‐resolved screening of mediators from different structural families (Figure 3) was conducted under otherwise standard conditions. We chose commercially available mediators that have already been used in various publications[^6^, ^16b^, ^21a^, ^21b^] to allow cross‐comparability to those protocols and to provide data for commercially accessible mediators. We focused on the class of stable *N*‐oxyl radicals, which are known to be the most powerful mediators for the investigated transformation.

**Figure 4:**
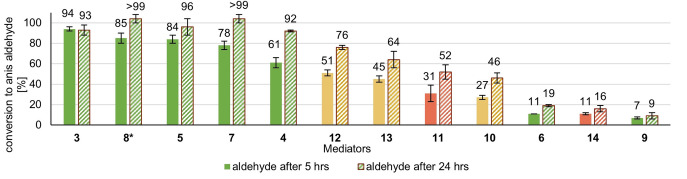
Time resolved mediator screening of commercially available mediators, reaction conditions: [Anis alcohol]=20 mM, [Mediator]=6 mM (0.3. equiv.), [*Tv*]=3 U/mL, 1 atm O_2_, rt, reaction time: 24 h, reaction monitoring *via* GC‐FID (internal standard method: methyl benzoate) after 5 and 24 h reaction time *measured *via* quant. NMR (internal standard method: 3,4,5‐trimethoxybenzaldehyde) after 5 and 24 h reaction time. All reactions were made in triplicates with standard deviation being indicated. Resulting mean values slightly exceeding 100 % were labeled as >99.

In Figure 4, the results of the mediator screening are shown: Conversions were measured after 5 and 24 h reaction times for all mediators and at additional time points for the best‐performing ones. Here, AZADOL (**3**) exhibits the highest mediator efficiency. Noteworthy, we did not directly utilize the *N*‐oxyl radical AZADO (**iii**) but generated the radical species (**iii**) *in situ* by applying the hydroxylamine AZADOL (**3**), due to the significant price difference between the two species (**iii**) and (**3**). However, based on our own experimental study (see the Supporting Information) and an overall understanding of the mechanism elucidated by Arends *et al*. goes, this has no significant impact on the outcome of the laccase‐mediated oxidation of alcohols.^[16a]^

Besides AZADOL, the TEMPO‐derivatives bearing a methoxy‐, hydroxy‐ and acetamido‐group (**8**,**5**,**7**) at the 4‐position are the most efficient mediators, outperforming TEMPO (**4**) itself. This is an interesting finding, as the previous literature from the Fabbrini and the Arends group suggested, that TEMPO (**4**) was at least equal or superior in the earlier studies.[^21a^, ^21b^] Prominently, methoxy‐TEMPO (**8**), our second‐best mediator achieving 85±5 % conversion to anisaldehyde (**2**) after only 5 h reaction time, was reported to only yield 26 % benzaldehyde after 24 h in the Arends publication of 2006 even though also a laccase from *T. versicolor* was used there.^[21a]^ Noteworthy, we analyzed the reaction progress of methoxy‐TEMPO (**8**) within our screening using quantitative NMR (all other mediators *via* GC‐FID), due to similar retention times of (**8**) and the substrate anis alcohol (**2**) in GC, even after several efforts of GC‐method optimization.

**Figure 5:**
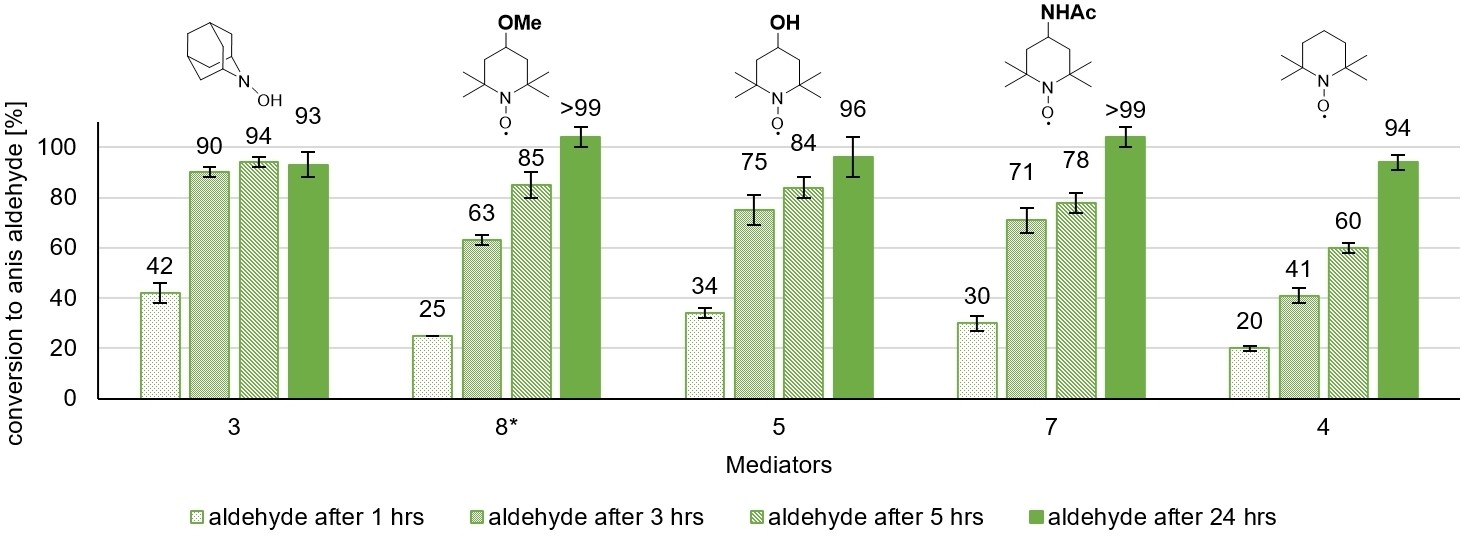
Time‐resolved mediator screening of 5 most efficient mediators providing two more timepoints, reaction conditions: [Anis alcohol]=20 mM, [Mediator]=6 mM (0.3. equiv.), [*Tv*]=3 U/mL, 1 atm O_2_, rt, reaction time: 24 h, reaction monitoring *via* GC‐FID (internal standard method: methyl benzoate) after 1, 3, 5 and 24 h reaction time *measured *via* quant. NMR (internal standard method: 3,4,5‐trimethoxybenzaldehyde) after 1, 3, 5 and 24 h reaction time with standard deviation being indicated. Resulting mean values slightly exceeding 100 % were labeled as >99.

*N*‐hydroxy benzotriazole (**12**, HBT) ranked the best mediator outside the TEMPO family, showing the same relative reactivity trends here as in a previous publication.[^6^, ^21b^] Violuric acid (**13**, VLA), ABTS (**11**), *N*‐hydroxy phthalimide (**10**, NHP), and Phenothiazine (**14**, PT) do not achieve high conversions, even after a 24 h reaction time, also consistent with the literature.[^21a^, ^21b^] Last, 4‐amino‐2,2,6,6‐tetramethyl‐piperidine‐1‐oxyl (**6**, amino‐TEMPO) and 2,2,6,6‐tetramethyl‐4‐piperidone (**9**, Oxo‐TEMPO) exhibit low conversions, despite being part of the TEMPO family. In the case of Oxo‐TEMPO (**9**), this is likely because the radical species is known to decompose at lower pH.^[27]^ This instability was later also underlined in our own CV‐measurements. The amino‐TEMPO, however is stable, and the reduced conversion seems to represent reduced mediator performance. Functionalization at the 4‐position can thus tune the reactivity of a TEMPO‐based mediator in both directions. To achieve a better comparability of the performance of the five highest‐performing mediators, we provide additional time points after 1 h and 3 h reaction time (Figure 5).

As before, AZADOL performs slightly better at all time points than the TEMPO derivatives **4**, **8**, **5**, and **7**. Considering all time points, methoxy‐TEMPO (**8**), hydroxy‐TEMPO (**5**) and acetamido‐TEMPO (**7**) perform somewhat similar but considerably better than the parent TEMPO (**4**). (Figure 5) As methoxy‐TEMPO (**8**), hydroxy‐TEMPO (**5**), and acetamido‐TEMPO (**7**) only perform slightly inferior to AZADOL (**3**); these are a competitive alternative when considering as AZADOL (**3**), as well as AZADO (**iii**), have only a relatively low solubility in aqueous solution and are significantly more expensive than the other mediators investigated in this screening.

### Cyclic voltammetry

For the mediator efficacy in laccase‐mediated oxidation, likely two factors are of importance: the redox potential (reflecting the ease with which it gets oxidized, but also its oxidative strength) and the redox stability over multiple cycles. Both of those factors can be determined *via* cyclic voltammetry (CV). In literature, the redox potentials are either given as anodic potentials E_a_ or as midpoint potentials E_1/2_. and are usually determined in buffered water with a pH of 4.7 or in acetonitrile.^[27a]^ We decided to determine the mediators′ midpoint potentials (E_1/2_) and redox‐stabilities exactly in the utilized buffer system that is required by *T. versicolor* (0.1 M NaOAc‐buffer, pH 4.5). The experimental redox potentials and redox stabilities of mediators **2**–**14** at pH 4.5 can be found in Table 2, sorted according to their midpoint potentials. Conversions after 5 h from the screenings as a surrogate for performance were included there. For a meaningful comparison, the investigated mediators are clustered according to the different mechanisms of alcohol oxidation. Thus, the redox potentials of the stable *N*‐Oxyl mediators **3**–**9** and **14** (Entries 1–7) are discussed separately from the *N*‐hydroxy compounds **10**, **12**, **13** (Entries 9–11).

**Table 2:** Redox potentials, redox stability and conversion after 5 h in anis alcohol oxidation of mediators (10 mM), measured in 0.1 M NaOAc‐buffer, pH 4.5, at a scan rate of 100 mV s^−1^. Entries 1–7: Stable *N*‐Oxyl radicals (ionic mechanism); Entry 8: ABTS (ET‐mechanism); Entry 9–11: *N*‐hydroxy type mediators (HAT‐mechanism).

Entry	Mediator	E_1/2_ [mV] *vs*. NHE	Conversion to **2** after 5 h [%]	Redox stability
1	Hydroxy‐TEMPO (**5**)	693	84±4	+
2	TEMPO (**4**)	696	61±5	+
3	Methoxy‐TEMPO (**8**)	702	85±5	+
4	Acetamido‐TEMPO (**7**)	704	78±4	∼
5	AZADOL (**3**)	774	94±3	+
6	Amino‐TEMPO (**14**)	806	11±0	+
7	Oxo‐TEMPO (**9**)	806	7±1	−
8	ABTS (**11**)	742 & 802	31±8	+
9	VLA (**13**)	987	45±3	+
10	NHP (**10**)	1168	27±3	+
11	HBT (**12**)	Not determinable^[a]^	51±3	^[a]^

[a] No distinct redox transition observed within the solvent window.

For the first group of TEMPO (**4**) and its derivatives (**5**, **7**, **8**, **14**, **9**), two groups are apparent: The first group comprises of compounds **4**, **5**, **8** and **7**. These mediators showed similar, good reactivities in the screening (Figure 5) and also exhibit very similar redox potentials (within 11 mV span).

**Figure 6:**
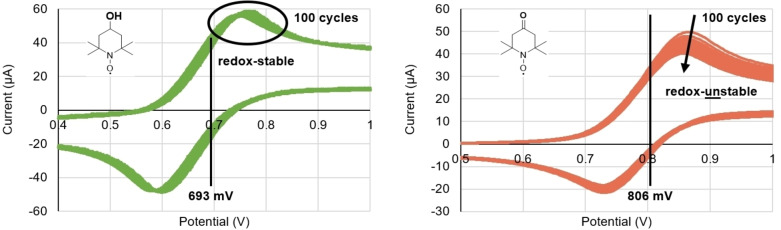
Exemplary cyclic voltammetry of Hydroxy‐TEMPO (5) and Oxo‐TEMPO (9). Hydroxy‐TEMPO remains stable under the investigated conditions, the peak of Oxo‐TEMPO drops off with increasing number of performed cycles (10 mM mediator, measured in 0.1 M NaOAc‐buffer, pH 4.5, at a scan rate of 100 mV s^−1^).

Furthermore, these compounds are all redox stable, except for acetamido‐TEMPO **7**, which showed slight decomposition in our CV experiments, although the residual activity makes it an overall well‐performing mediator (Figure 6, left for an example). The second group of TEMPO derivatives is amino‐TEMPO (14) and Oxo‐TEMPO (9), for which we both obtained an E_1/2_ value of 806 mV vs. the normal hydrogen electrode (NHE). This is around 100 mV higher than for the previous group (Entry 1–4). Interestingly, both 9 and 14 show significantly lower reactivity towards anis alcohol (**1**), compared to the first group, likely due to different reasons as judged by the CVs.

Firstly, Oxo‐TEMPO is not redox‐stable (Figure 6, right). It is literature known to undergo an abstraction of its α‐hydrogens in its oxidized state, the oxoammonium ion, ultimately leading to bond‐breaking next to the reactive *N*‐oxyl radical functionality and, thereby, inactivation of the mediator.^[28]^ On the other hand, although amino‐TEMPO (**14**) is redox‐stable, it is not an efficient mediator in both our system as well as in literature, potentially due to its higher redox potential.^[21a]^ A greater collection of measured cyclic voltammograms are enclosed in the Supporting Information data.

However, the redox potential of AZADOL (**3**, Entry 5) is also significantly higher (≈70 mV) than the ones of Group 1 (Entries 1–4). Still, AZADOL (**3**) exhibits superior catalytic efficiency than the TEMPO derivatives. We hypothesize that the reason for this discrepancy are the kinetic benefits AZADOL has because of its reduced steric hindrance compared to the TEMPO family.^[16b]^ Concluding, despite the fact that redox stability is a crucial parameter for the performance of a mediator, the redox potential is only one factor contributing to a mediator‘s performance. Other factors, such as steric hindrance and its effect on kinetic parameters, such as e. g. the electron self‐exchange reaction constant, also seem to be important.[^16b^, ^29^]

### Investigation of the key concentrations of the laccase‐mediated oxidation of anis alcohol

**Scheme 4:**
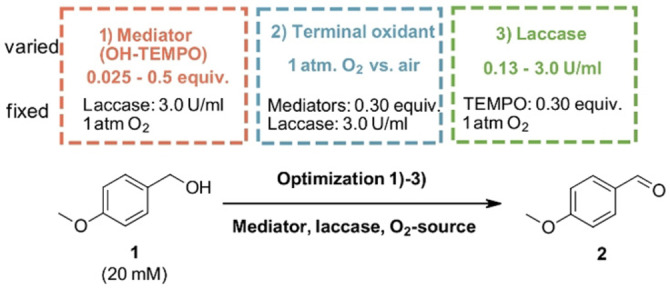
Overview of our optimization experiments evaluating 1) mediator concentration 2) laccase activity and 3) application of oxygen *vs*. air.

With our optimized laccase/mediator pairs at hand, we aimed at optimizing relative concentrations towards an (economically) efficient overall oxidation. This is particularly relevant at larger scales and for industrial applications. The applied conditions for the mediator and laccase screenings were carried out with a relatively high mediator loading of 0.3 equiv. compared to the substrate and 3.0 U/mL laccase. However, in the light of optimizing those conditions for potent laccase/mediator pairs, the following three parameters were evaluated. 1) Mediator concentration, 2) Activity of laccase and 3) O_2_ *vs*. air as terminal oxidant. (Scheme 4).

Concerning the mediator concentration, we addressed the question: Which mediator loading leads to the best cost‐benefit ratio? Because on the one hand, if too much mediator is added, without achieving faster conversions, mediator, hence money, is wasted. On the other hand, if too little mediator is applied, the reaction time is increased, wasting reactor time and, thus again, money.

**Figure 7:**
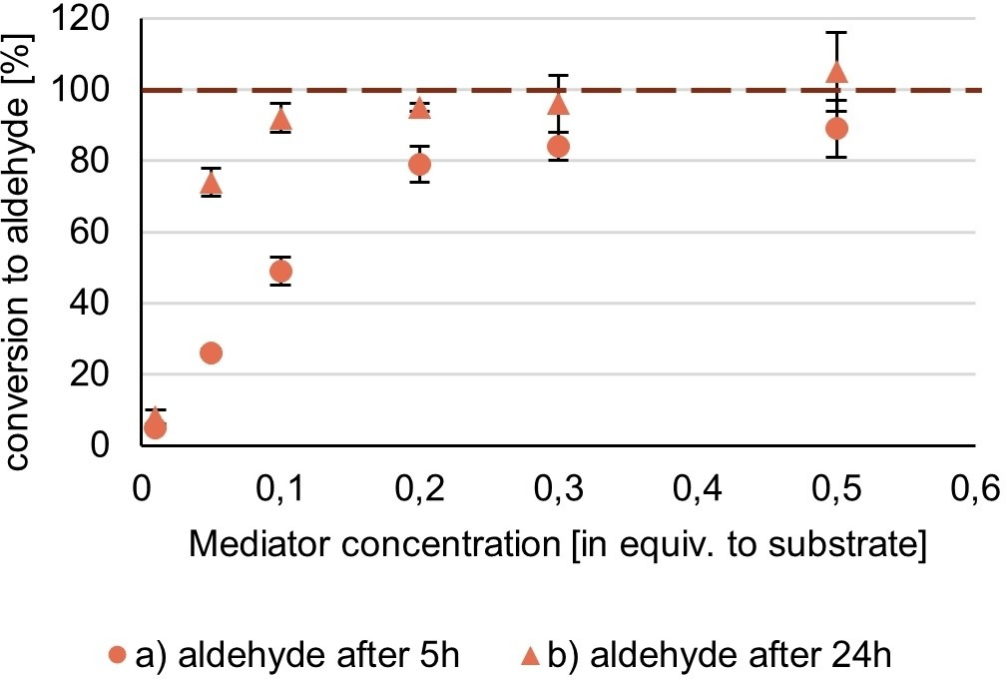
Conversion to anis aldehyde after a) 5 h reaction time b) 24 h reaction time; conditions: [Anis alcohol]=20 mM, [Hydroxy‐TEMPO **5**]=10 mM (0.5 equiv.), 6 mM (0.3 equiv.=30 mol %), 4 (0.2 equiv.=20 mol %), 2 mM (0.1 equiv.=10 mol %), 1 mM (0.05 equiv.=5 mol %) 0.2 mM (0.025. equiv.=2.5 mol %), activity (*T. versicolor*)=3 U/mL, 1 atm O_2_, rt, reaction time: 24 h, reaction monitoring *via* GC‐FID (internal standard method: methyl benzoate) after 5 h and 24 h reaction time.

To evaluate the optimal mediator loading for a given substrate concentration and enzyme activity, we varied the concentration of Hydroxy‐TEMPO (**5**), an efficient mediator in the previous screening, from 0.50 to 0.025 equiv. The following diagram (Figure 7) shows the conversion to anisaldehyde for the different loadings of Hydroxy‐TEMPO (**5**) (0.5, 0.3, 0.2, 0.1, 0.05, 0.025 mol %) after a) 5 h and b) 24 h.

A strong correlation between the mediator concentration and the conversion to anisaldehyde was found (Figure 7), both after 5 h and after 24 h. As depicted by the orange horizontal line in Figure 7, after 5 h with 0.2 equiv. and after 24 h, even with 0.1 equiv. mediator, complete conversion, was achieved, below 0.1 equiv. yields dropped quickly. Therefore, mediator loadings from 0.3 to 0.1 equiv. are the most economical, depending on the goal: very short reaction times or fast completion of reaction. Noteworthy, the observed data resembles a reaction with Michaelis‐Menten (MM) kinetics, however, the respective parameters cannot be derived from it. The initial enzymatic oxidation of TEMPO does follow Michaelis‐Menten kinetics and was directly determined in the literature for TEMPO and laccase from *Trametes versicolor*. However, the same paper showed that the second step (chemical oxidation) is rate determining, thus preventing the deduction of MM from the anis aldehyde conversion.[^16a^, ^30^]

The second important factor of the laccase‐mediated oxidation we investigated is the applied form of the stoichiometric oxidant: oxygen, which re‐oxidizes the reduced laccase upon formation of water (Scheme 1). This step in our enzymatic reaction is reported to be the fastest, e. g., for *T. versicolor* k_cat_ >10^3^ have been measured.^[30]^ Thus, replacing pure O_2_ with more benign air should be feasible.

**Figure 8:**
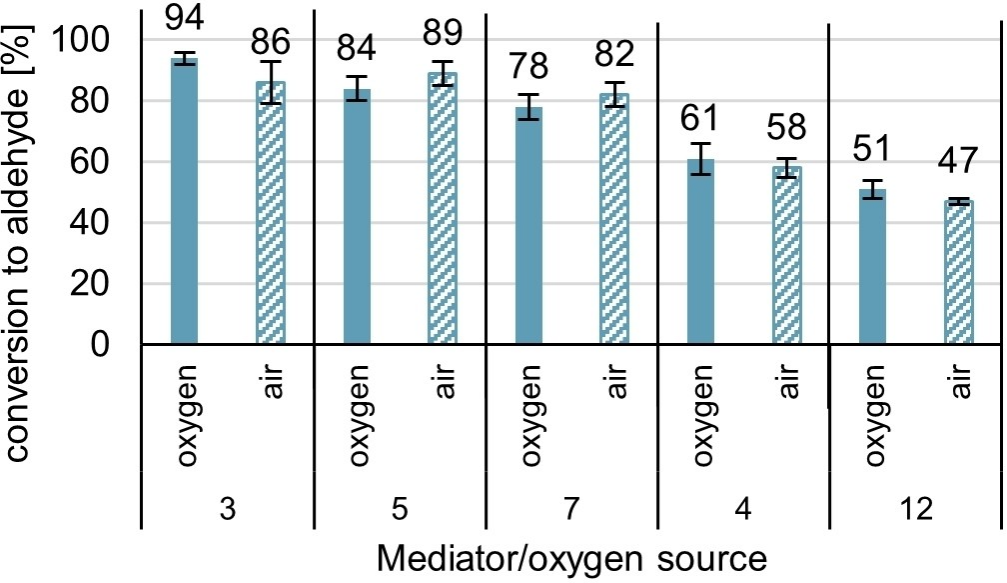
Comparison between two different sources of stoichiometric oxidant O_2_: pure oxygen and air. Conditions: [Anis alcohol]=20 mM, [TEMPO **4**]=6 mM (0.3 equiv.) [*Tv*]=3 U/mL, 1 atm O_2_/air, rt, reaction time: 5 h, reaction monitoring *via* GC‐FID (internal standard method: methyl benzoate) after 5 h reaction time with standard deviation being indicated.

The comparison of the conversions to aldehyde [%] after 5 h for various mediators was measured, applying 1 atm. O_2_ and 1 atm air, representing a fivefold reduction of partial pressure of O_2_. The results can be found in Figure 8 and Table 3.

**Table 3:** Overview of mediators employed for air/oxygen comparison.

Entry	Number	Mediator
1	**3**	Methoxy‐TEMPO
2	**5**	Hydroxy‐TEMPO
3	**7**	Acetamido‐TEMPO
4	**4**	TEMPO
5	**12**	1‐Hydroxy benzotriazole

As the data from Figure 8 suggests, there is no significant difference in anis alcohol conversion between using O_2_ or air as an oxidant. This not only parallels with the reported experimentally measured, very high k_cat_ value of these conversions but means that the cheapest, most abundant oxidant source, air, can be used in a laccase mediated oxidation without reactivity losses. On a large, industrial scale, this means that the potential safety hazard of pure oxygen can be replaced by benign, cheap, and abundant air, at least as long as the phase transfer from the gas phase to the solution is not limiting. We enclosed further information about the closely measured oxygen levels throughout the laccase‐mediated oxidation of anis alcohol in the Supporting Information file.

**Figure 9:**
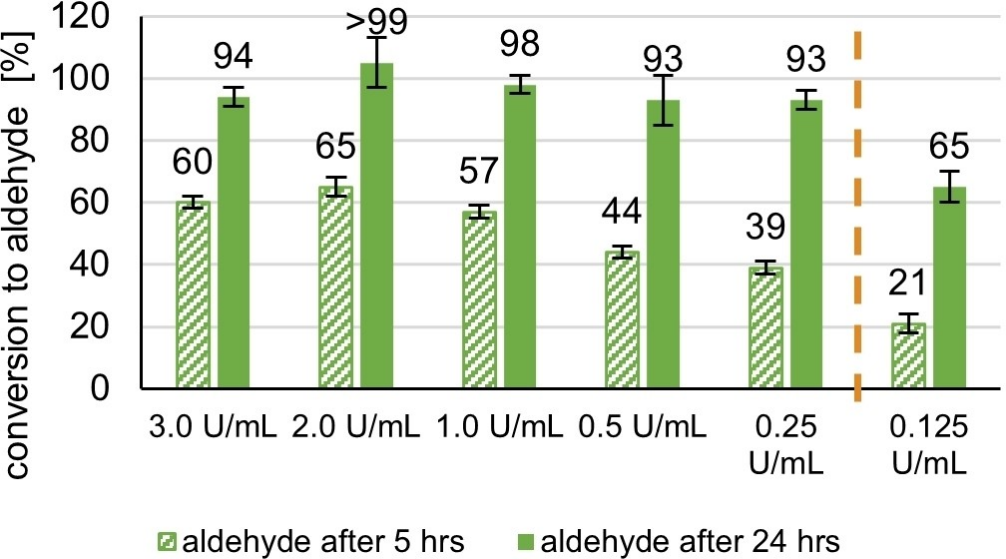
Laccase activity screening: conditions: [Anis alcohol]=20 mM, [TEMPO **4**]=6 mM (0.3 equiv.) [*Tv*]=3.0, 2.0, 1.0, 0.50, 0.25, 0.125 U/mL, 1 atm O_2_, rt, reaction time: 5 h, reaction monitoring *via* GC‐FID (internal standard method: methyl benzoate) after 5 h reaction time with standard deviation being indicated. Resulting mean values slightly exceeding 100 % were labeled as >99.

The last factor we addressed was the laccase activity. A laccase optimization study was carried out, running the oxidation under 3, 2, 1, 0.5, 0.25, 0.1 U/mL and – using TEMPO (**4**) (0.3 equiv.) as standard benchmarking mediator. Figure 9 shows the conversions to anisaldehyde after 5 and 24 h. It becomes evident that at least for the substrate anis alcohol, a commonly used activity of 3.0 U/mL[^21a^, ^21b^] is more than enough to achieve fast conversions: Interestingly, with laccase from *T. versicolor*, with concentrations down to 0.25 U/mL – a 12 fold reduction – similar conversions are achieved; below this, there seems to be is a significant cut‐off of the system‘s efficiency (Figure 9, orange bar) best seen at the 24 h timepoint.

We hypothesize, that this cut‐off occurs at the point in which the rate determining step of the system changes: If there is enough laccase present, the slowest reaction step is the oxidation reaction between oxoammonium and alcohol.^[16a]^ However, laccase concentrations <0.25 U/mL are not enough to quickly oxidize the *N*‐oxyl to yield the actual oxidant, the oxoammonium species. Under the assumption that the oxidation of the material of interest does not become rate determining, these ratios should even be transferrable to other reactions. In any case, quite generally, determining such cut‐off concentration allows substantial saving on laccase without sacrificing much oxidation efficiency.

## Conclusion

An operationally facile, quantitative anis alcohol model system was established to explore the multivariate, highly complex system of the laccase mediated oxidation of alcohols in a time‐resolved manner.

With this assay, first, the efficiency of nine laccases was compared, presenting laccases with high redox potentials, such as from *T. versicolor*, as clearly superior.

Furthermore, a mediator screening was carried out using the commercially available, high redox‐potential laccase from *T. versicolor*. Among the thirteen tested, the mediators achieving the highest yields of anis aldehyde (**2**) were AZADOL (**3**) and methoxy‐TEMPO (**8**), hydroxy‐TEMPO (**5**), and acetamido TEMPO (**7**), all outperforming TEMPO (**4**). It was shown that methoxy‐TEMPO (**8**) is significantly higher performing than compared to prior publications.

The redox potentials (midpoint potentials E_1/2_) were measured for most mediators of our screening to relate to the observed relative reactivities. Efficient TEMPO derivatives **5**, **7**, **8** and TEMPO (**4**), had very similar redox potentials in the range of 694–704 mV *vs*. the NHE. AZADOL (**3**) exhibits a significantly higher midpoint potential of 774 mV, although still showing the most catalytic efficiency in the oxidation of anis alcohol (**1**), likely due to less steric hindrance, which aligns with what Zhu *et al*. suggested in 2014.

Last, we investigated the effect of the mediator and laccase concentration: We found a strong correlation between mediator concentration and the anis aldehyde (**2**) consumption. The most economical mediator loading in our model system was between 0.1–0.2 equivalents compared to the substrate. Additionally, we showed that O_2_ can be replaced by air with similar conversions to **2**, for all tested mediators at the scale investigated.

Considering laccase concentration, we can propose a drastic decrease of laccase activity (as main driver of cost) to 8 % of the original 3 U/mL, while preserving most of the system‘s efficiency.

We are convinced that our comparative study will be a great resource for both people entering the field of mediator‐assisted laccase‐catalyzed alcohol oxidations. Further, we hope it will serve as a common base for us and other to further improve ideal mediator structure and the general interplay of all components of this attractive but complex setting for biocatalytic alcohol oxidations.

## Experimental Section

**Materials**: All chemicals were used directly from commercial sources and used without further purification. Laccase from *T. versicolor* (0.510 U/mg) was purchased from Sigma Aldrich as a lyophilized powder, light brown. Laccase A (0.173 U/mL), F (1.39 U/mg, from *T. versicolor*), U (13.2 U/mg), and PP (0.478 U/mg), were gifted from ASA‐Spezialenzyme as lyophilized powders. Laccase from *T. hirsuta* (232 U/mL), *Bacillus Spore coat* Laccase (157 U/mL), and from *Streptomyces ipomoeae* (6.4 U/mL) were provided by the Gübitz group (University of Natural Resources and Life Sciences Vienna). The mediators were purchased from TCI and Sigma Aldrich and were used directly without further purification.

**Instruments**: GC analysis was carried out on a Thermo Finnigan Focus GC/DSQ II equipped with a standard capillary column (BGB5, 30 m×0.25 mm ID, 0.50 μm film) with an FID detector. Carrier gas: helium, injector: 230 °C; column flow: 2.0 mL/min; method for quantification: 80 °C (0 °C/min, 1 min)→80–280 °C (40 °C/min, 7 min). NMR spectra were recorded at 297 K in the solvent indicated with an Avance UltraShield 400 and an Avance III HD 600 spectrometer. All spectra were calibrated to the solvent residual peak. Chemical shifts (δ) and coupling constants (*J*) were expressed in ppm and Hz, respectively.

**Cyclic voltammetry**: Cyclic voltammetry (CV) experiments were carried out with an Metrohm Autolab potentiostat (PGSTAT204), in a 0.1 M sodium acetate buffer at pH 4.5, with a platinum working electrode (planar disk, Ø 2 mm), a platinum counter electrode (wire, Ø 0.5 mm) and an Ag/AgCl reference electrode at a scan rate of 100 mV s^−1^. The concentration of the substrate was 10 mM.

**Enzyme activity measurement**: The enzyme activity was determined photometrically at 25 °C using ABTS as substrate. ABTS 11 (50 μL of a 0.01 M solution) was added to a well plate. Then, 170 μL of an enzyme solution (0.005–0.05 mg/mL) was added. The absorbance change was recorded at 405 nm with 5‐s pre‐read shake ϵ(ABTS)=36.8 l*mmol^−1^*cm^−1^ at 420 nm. All measurements were carried out in triplicates.

### Synthetic procedures

**The standard procedure of laccase‐mediated oxidation of anis alcohol**: Mediator (12.0 μmol, 0.3 equiv.) was placed in an 8 mL glass vial filled with 0.1 M sodium acetate buffer (pH 4.5), and the vial was purged with oxygen/air for 5 min. After adding 40 μL of a 1 M anis alcohol (**1**) stock solution (5.5 mg, 40 μmol, 1.0 equiv., ACN), laccase (*T. versicolor*, 3.0 U/mL, dissolved in buffer) was added to the vial, resulting in 2 mL of reaction mixture. The vial was connected to a balloon filled with oxygen/air. The mixture was stirred at room temperature.

For GC‐FID analysis, samples (200 μL) were taken at given times, at least t_0_ (initial time, 0 h)_,_ t_1_ (5 h), and t_2_ (24 h).

*Workup of GC samples*: The reaction mixture (200 μL) was extracted twice with ethyl acetate (EtOAc) supplemented with 1 mM methyl benzoate (IS, 200 μL) and dried over Na_2_SO_4_. Then 100 μL were diluted with 200 μL 1 mM methyl benzoate in EtOAc and filled in a GC vial with 0.1 mL inlet.

*Workup for quant. NMR*: The reaction mixture (900 μL) was extracted with 2×0.5 mL of CDCl_3_ supplemented with 20 mM 3,4,5‐trimethoxy benzaldehyde (IS). As emulsions were occurring during extraction, the biphasic mixture was centrifuged. The extract was dried over Na_2_SO_4_ and submitted to NMR. If necessary, CDCl_3_ was added when there was less than 0.6 mL in the NMR tube. The quantification was calculated *via* a literature‐known technique.^[31]^

## Conflict of interest

The authors declare no conflict of interest.

1

## Supporting information

As a service to our authors and readers, this journal provides supporting information supplied by the authors. Such materials are peer reviewed and may be re‐organized for online delivery, but are not copy‐edited or typeset. Technical support issues arising from supporting information (other than missing files) should be addressed to the authors.

Supporting Information

## Data Availability

The data that support the findings of this study are available from the corresponding author upon reasonable request.
