# Supplementary material for: An Organic Chemist's Guide to Mediated Laccase Oxidation
Source: Chembiochem. 2022 Oct 18;23(23):e202200411. doi: 10.1002/cbic.202200411 (PMC10092592; doi:10.1002/cbic.202200411)
Supplement: Supplementary file 1 — Supporting Information [file CBIC-23-0-s001.pdf]

ChemBioChem

Supporting Information

An Organic Chemist's Guide to Mediated Laccase Oxidation

Katharina Obleser[+], Hubert Kalaus[+], Bernhard Seidl, Martin Kozich, Christian Stanetty,* and Marko D. Mihovilovic

Table of Contents

A.1. Materials & Methods 2

A.2. Control Experiments 3

A.3. Determination of Influence of Redox-State of Mediators in the Laccase-Mediated Oxidation of Anis Alcohol 4

A.4. Synthesis of Mediators 5

A.4.1. Synthesis of 1-hydroxy-4-methoxy-2,2,6,6-tetramethylpiperidine **15** 5

A.4.2. Synthesis of 4-methoxy-2,2,6,6-tetramethyl-1-oxopiperidinium tetrafluoroborate **25** 6

A.5. Quantification of Screenings *via* Quantitative NMR for Anis Aldehyde System 7

A.6. Investigations of the Stoichiometric Oxidant 8

A.7. Cyclic Voltammogramms 10

A.7.1. AZADOL (3) 10

A.7.2. TEMPO (4) 11

A.7.3. Hydroxy-TEMPO (5) 12

A.7.4. Acetamido-TEMPO (7) 13

A.7.5. Methoxy-TEMPO (8) 14

A.7.6. Oxo-TEMPO (9) 15

A.7.7. NHP (10) 16

A.7.8. ABTS (11) 17

A.7.9. HBT (12) 18

A.7.10. VLA (13) 19

A.7.11. Amino-TEMPO (14) 20

A.8. Literature 21

A.1. Materials & Methods

Reagents and solvents

All chemicals were used directly from commercial sources and used without further purification. Laccase from *Trametes versicolor* (0.510 U/mg) was purchased from Sigma Aldrich as a lyophilized powder, light brown. Laccase A (0.173 U/ml), F (1.39 U/mg, from *Trametes versicolor*), U (13.2 U/mg), and PP (0.478 U/mg), were gifts from ASA-Spezialenzyme as lyophilized powders. Laccase from *Trametes hirsuta* (232 U/ml), Bacillus spore coat laccase (157 U/ml), and from *Streptomyces ipomoeae* (6.4 U/ml) were provided by the Gübitz group (University of Natural Resources and Life Sciences Vienna). The mediators were purchased from TCI and Sigma Aldrich and were used directly without further purification. Unless remarked differently, water-free solvents were used directly from the in-house solvent drying plant (PureSolv EN1-4), or commercial sourced with a water-free specification stored in bottles with a septum and over a molecular sieve.

Optical rotation

To measure the optical rotation of the chiral compounds the modular circular polarimeter *500 (MCP 500)* from *Anton Paar* with a 100 mm long cuvette of a 3 mm diameter was used. The measurements took place at 20 °C and a wavelength of 589 nm with c = 1.0 for most substances as indicated at the respective protocols.

UV/VIS measurements

UV/VIS measurements were performed on a plate reader *Zenyth 3100* from *Anthos*.

Oxygen-meter

Oxygen saturation measurements were carried out with Firesting-O_2. The software for processing was Pyro Workbench.

Thin-layer chromatography (TLC)

TLC analysis for reaction monitoring was performed on silica gel 60 F254-plates. The spots were visualized *via* UV light (254 nm) followed by staining the plates with anisaldehyde solution (180 ml EtOH, 10 ml anisaldehyde, 10 ml H_2SO_4 conc., 2 ml AcOH), potassium permanganate solution (3.0 g $KMnO_4$, 20.0 g K_2CO_3, 250 mg KOH, 300 ml H_2O) or cerium molybdate solution (“Mostain”, 21 g $(NH_4)_6Mo_7O_{24}\cdot4\,H_2O$, 1 g $Ce(SO_4)_2$ 31 ml H_2SO_4 conc., 500 ml H_2O).

Nuclear Magnetic Resonance (NMR)

NMR spectra were recorded on a *Brucker Avance UltraShield 400* spectrometer (400 MHz machine) or a *Brucker Avance III HD 600* spectrometer (600 MHz machine). Spectra were calibrated to the solvent residual signal[1]. Coupling constants (J) are given in Hz and chemical shifts (δ) in ppm. Assignments are based on COSY, HSQC and HMBC spectra and follow IUPAC nomenclature. Determined according to phase-sensitive experiments (HSQC, APT, DEPT or DEPTQ) for ^{13}C-NMRs virtual coupling patterns are given for (s, d, t, q for CH_3, CH_2, CH, C).

For *in-situ* reduction *N*-oxyl radicals in the NMR tube, addition of 55 mg of phenylhydrazine in 0.6 ml $CDCl_3$ was utilized, following a modified literature protocol.[2]

Gas Chromatography (GC)

GC analysis of anisaldehyde oxidations was carried out on a *Trace* Dual GC, equipped with a standard capillary column (BGB5, 30 m x 0.25 mm ID, 0.50 μm film) with an FID detector. Carrier gas: helium, injector: 230 °C; column flow: 2.0 ml/min; method for quantification: 80 °C (0°C/min,1 min) → 80–280 °C (40 °C/min, 7 min). The software for processing used was Chromeleon.

Melting points

Melting points were recorded on a Kofler-type *Leica Galen III and are uncorrected*

Enzyme activity measurement

The enzyme activity was determined photometrically at 25°C using ABTS as substrate. Into a well plate, 50 µL of a 0.01 M ABTS solution and then, 170 µL of a 0.05 mg/ml solution of laccase was added. The absorbance change was recorded at 405 nm with 5-sec pre-read shake (ε(ABTS)=36.8 $l \cdot mmol^{-1} \cdot cm^{-1}$ at 420 nm). The activity was determined from the linear range of the curve. Equation 21 describes the slope of the linear section of the curve:

$$Slope = \frac{dA}{dt} = \frac{d(\varepsilon\, c\, d)}{dt}\ [\frac{1}{min}] \tag{21}$$

A…absorption, d…pathlength [0.61 cm], c…concentration [mol/l]

The calculation of the enzyme activity A [$\mu molmin^{-1}ml^{-1} = Uml^{-1}$] was carried out according to equation 22.

$$A = (Slope_{test} - Slope_{blank}) \frac{1}{\varepsilon\, d} \cdot \frac{Volume_{total}}{Volume_{enzyme}} \cdot dilution\ factor \tag{22}$$

A.2. Control Experiments

The set-up of our model system using anisaldehyde is depicted in Scheme 1 and the control experiments in Table 1:

Anis Alcohol Model System

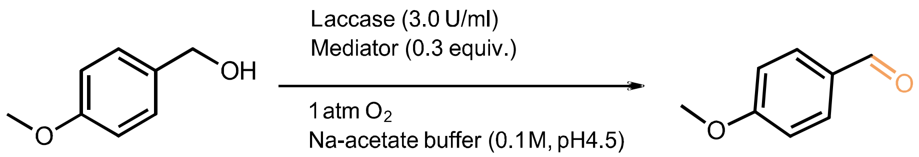

xx (20 mM) **xx**

Scheme 1: Reaction conditions of the laccase-mediated oxidation of anis alcohol as the model substrate

Table 1: control experiments: reaction conditions: [Anis alcohol] = 20 mM, [TEMPO] = 6 mM (0.3. equiv.), [*T.versicolor*]=3.0 U/ml, 1 atm O_2, rt, reaction time: 24 h, reaction monitoring *via* GC-FID (internal standard method: methyl benzoate) 24h reaction time

Entry	Control experiment	Conversion after 24 hrs
1	Laccase (no TEMPO)	0%
2	TEMPO (no laccase)	0%
3	Anis aldehyde + TEMPO + laccase+ O_2	0%
4	Laccase+ TEMPO	94±3%

A.3. Determination of Influence of Redox-State of Mediators in the Laccase-Mediated Oxidation of Anis Alcohol

Accompanying our mediator screening, we conducted a short study on the influence of oxidation state on an *N*-oxyl based mediator on its reactivity in the laccase-mediated oxidation of anis alcohol. According to the mechanism of the laccase-mediated oxidation of benzyl alcohol, elucidated by Tromp *et al.*[3], this should not lead to differences in mediator performance. In order to conduct this investigation, methoxy-TEMPO **(8)** was converted into its respective oxidized and reduced forms acordding to literature protocols[4] (Scheme 2). All species **(15**, **8,** and **16**) were screened *via* our anis alcohol system.

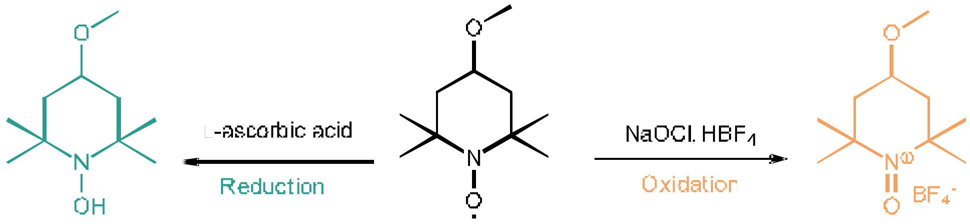

15 **8** **16**

Scheme 2: Conversion of methoxy-TEMPO (**8**) in the reduced form (**15**) and the oxidized form **(16).**

As shown in Table 2, there is no significant difference in mediator performance between the three possible oxidation states. This implies that similar results can be expected regardless of which species is provided. This was reassuring, as we utilized AZADOL **(3)** instead of even more expensive AZADO **(iii)** in our following mediator screening. Additionally, this information is beneficial for those who develop synthetic routes towards new *N*-oxyl mediators, as those can be simplified by targeting the most accessible or stable oxidation state.

Table 2: Conversions to anis aldehyde after 5 and 24h applying methoxy- TEMPO (**8**) in different oxidation states, reaction conditions: [Anis alcohol]=20 mM, [Mediator]=6 mM (0.3. equiv.), [*Tv*]=3 U/ml, 1 atm O_2, rt, reaction time: 24 h, reaction monitoring *via* quant. NMR (internal standard method: 3,4,5-trimethoxybenzaldehyde) after 5 and 24 h reaction time.

Species	Conversion after 5h [%]	Conversion after 24h [%]
Oxoammonium **16**	80%	100%
Hydroxylamine **15**	93%	100%
N-Oxyl radical **8**	93%	100%

A.4. Synthesis of Mediators

A.4.1. Synthesis of 1-hydroxy-4-methoxy-2,2,6,6-tetramethylpiperidine **15**

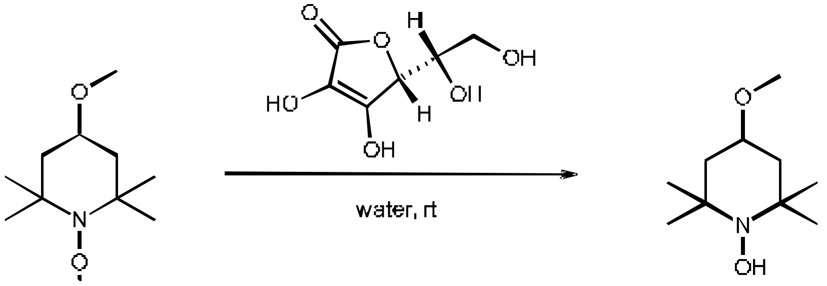

8 **15**

Procedure: Reduction

Following a modified literature protocol [4a] ascorbic acid (821 mg, 4.64 mmol, 1.60 equiv.) in water (10 ml) was added to a solution of the *N*-oxyl **11** (502 mg, 2.91 mmol, 1.00 equiv.) in water (40 ml). The red solution decolorized instantaneously, and TLC analysis (LP: EtOAc 1:4) indicated complete conversion. It was then extracted with Et_2O (5 × 50 ml), the ether extract was dried (Na_2SO_4) and evaporated. The target compound was isolated as colorless crude solid **15** (377 mg) with 99% purity according to ^{1}H-NMR analysis.

**Yield:**	377 mg (75 %)
**Appearance:**	colorless solid
**Melting point**	39 - 40 °C
**R_f-value**	0.30 (LP: EtOAc 1:4)
**NMR:**	^{1}H NMR (400 MHz, Chloroform-*d*) δ 3.47 (tt, *J* = 11.2, 4.1 Hz, 1H, CH), 3.34 (s, 3H, OCH_3), 1.95 (s, 2H, CH_2), 1.40 (t, *J* = 11.9 Hz, 2H, CH_2), 1.21 (s, 6H, 2× CH_3), 1.16 (s, 6H, 2× CH_3).
	^{13}C NMR (151 MHz, Chloroform-*d*) δ 71.9 (d, **C**H), 55.8 (q, O**C**H_3), 44.4 (2 × t, **C**H_2), 32.3, 20.6 (q, 4 × **C**H_3).

Spectral data in accordance with the literature. [5]

A.4.2. Synthesis of 4-methoxy-2,2,6,6-tetramethyl-1-oxopiperidinium tetrafluoroborate **25**

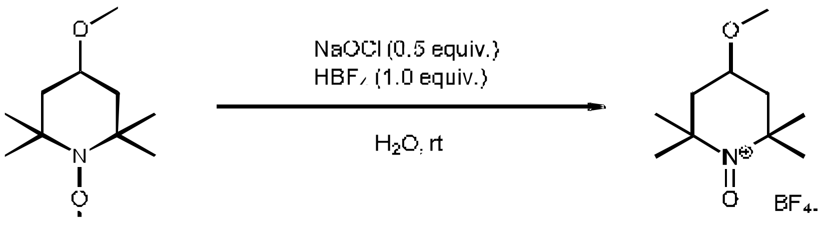

Procedure: Oxidation

Following a modified literature protocol[4b], methoxy-TEMPO **11** (2.00 g, 10.7 mmol, 1.00 equiv.) was dispersed in H_2O (6.4 ml). At room temperature, HBF_4 (48%, 1.96 g, 10.7 mmol, 1.00 equiv.) was slowly added dropwise over a period of 10 min. After the orange slurry turned to a yellow color, NaOCl (2.95 g, 5.37 mmol, 0.50 equiv.) was added over 15 min at 0 °C and stirred for an additional 1 h at 0 °C. The reaction mixture was filtered, and the yellow crystalline precipitate was washed with ice-cold 5% $NaHCO_3$ (4 ml), water (8 ml), and ice-cold Et_2O (80 ml) to yield the product as the bright yellow solid. The solid was dried in a desiccator overnight, yielding 1.77 g of a solid. ^{1}H-NMR indicated that the product was 99% pure. An aliquot of 1.00 g of the crude product was recrystallized in 14 ml H_2O yielding 780 mg (78% recovery) of target product **16** in yellow needles.*

**Yield:**	1.77 g (60 %,)
**Appearance:**	Yellow solid
**Melting point**	Decomposition at 97 °C
**NMR:**	^{1}H NMR (600 MHz, TFA) δ 4.59 (tt, J = 7.2, 4.5 Hz, 1H, CH), 3.67 (s, 3H, OCH_3), 3.05 (dd, J = 14.8, 4.5 Hz, 2H, C**H**H), 2.90 (dd, J = 14.8, 7.2 Hz, 2H, C**H**H), 1.86 – 1.78 (m, 12H, 4× CH_3).
	^{13}C NMR (151 MHz, TFA) δ 72.6 (td, **C**H), 58.2 (s, O**C**H_3), 44.5 (d, 2× CH_2), 32.6, 31.9 (d, 2× 2× CH_3).

Spectral data in accordance with the literature. [6]

*The recrystallization of an already pure product (according to ^{1}H-NMR) was carried out to determine whether the decomposition at 97°C still takes place for recrystallized product **25**, which it did.

A.5. Quantification of Screenings *via* Quantitative NMR for Anis Aldehyde System

The NMR samples in $CDCl_3$ supplemented with 20 mM 3,4,5-trimethoxy benzaldehyde **17** as internal standard were quantified *via* equation 1.

$$m_x = P_{std} \cdot \frac{MW_x}{MW_{std}} \cdot \frac{nH_{std}}{nH_x} \cdot \frac{m_{std}}{P_x} \cdot \frac{A_x}{A_{std}} \tag{1}$$

m_x, m_{std}…masses of analyte x and internal standard std in g
MW_x and MW_{std}…molecular weights in g/mol
P_x and P_{std}…purities (=1)
nH_x and nH_{std}…number protons generating the selected signals for integration
A_x and A_{std}…areas for the selected peak

Table 3 shows the selected ^{1}H-NMR signals used for quantification in the anis aldehyde system. The results using equation 1 of those signals were then averaged for each compound to lead to an average mass of analyte x. One example of an ^{1}H-NMR of an anis alcohol is shown in Figure 1.

Table 3: selected 1H-NMR signals for quantification of anis alcohol model system

Compound	δ [ppm]	Multiplicity	Number of H-atoms (nH)
**Internal standard (17)**	9.89	s	1
	7.14	s	2
**Anis alcohol (1)**	7.3	d J(8.55 ppm)	2
	7.42	d	2
	6.89	d J(8.55 ppm)	2
	4.63	s	2
**Anis aldehyde (2)**	9.87	s	1
	7.84	d J(8.69 ppm)	2
	7.01	d J(8.73 ppm)	2

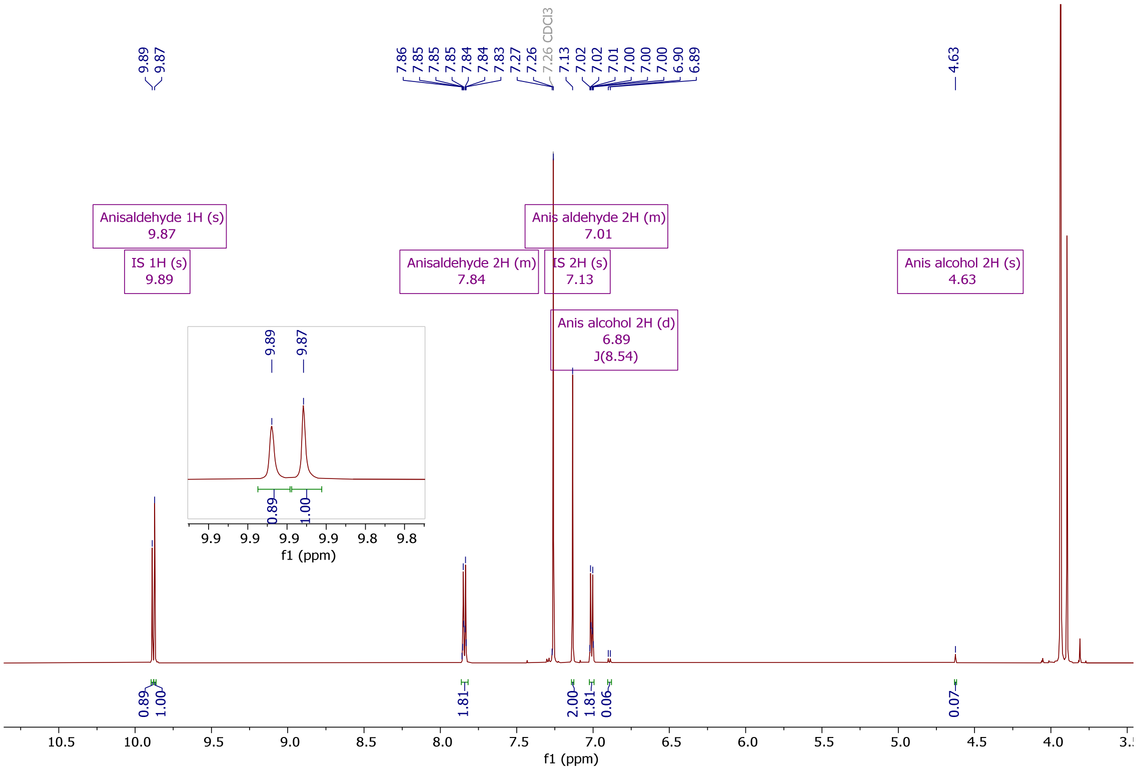

Figure 1: Example of an [1]*H-NMR used for quantification of a laccase-mediated oxidation of anis alcohol (Mediator: methoxy -TEMPO, reaction time: 5 h). Standard conditions.*

A.6. Investigations of the Stoichiometric Oxidant

Another crucial factor determining the efficacy of a laccase-mediator system is the accessibility of the stoichiometric oxidant, i.e. molecular oxygen. According to procedures usually found in the literature, the reaction mixture is saturated *via* an external oxygen source (usually an oxygen balloon on a syringe that is put into the solution). After saturation, then the oxygen source is connected *via* the gas phase to the reaction mixture (e.g., the balloon is placed on a septum of a closed vial or flask). We were particularly interested in following the oxygen saturation of the reaction mixture over time using the standard protocol for our anis alcohol oxidation. We utilized an oxygen meter to follow the oxygen concentration during a laccase-TEMPO mediated oxidation of anis alcohol according to our standard screening conditions (Scheme 3), as shown in Figure 2.

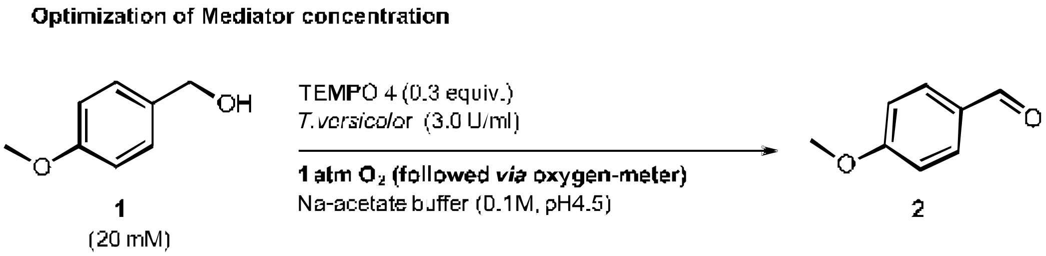

Scheme 3: Reaction conditions for the investigation of the oxygen saturation on the reaction mixture of a laccase-mediated oxidation of anis alcohol ***5***

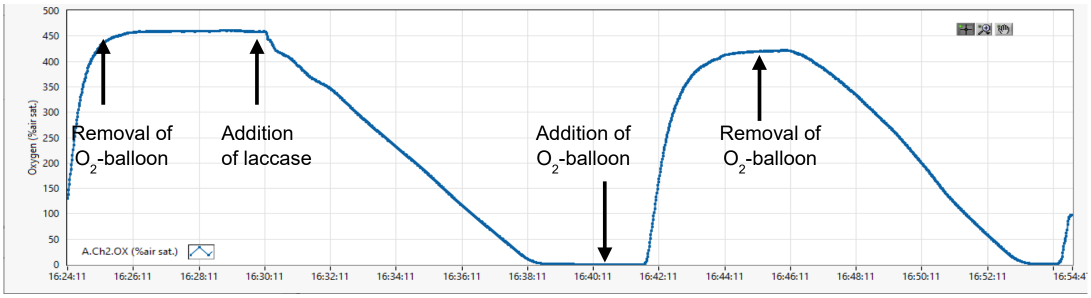

*Figure 2: Time/oxygen saturation diagram of a laccase-mediated anis alcohol **5** oxidation reaction (standard conditions, mediator: TEMPO **2**)*

The diagram in Figure 2 shows the oxygen saturation in the reaction mixture over time. The first drastic increase of oxygen concentration arises from the addition of an oxygen balloon. Regarding the O_2-intake, it is very common to saturate reaction mixtures of several milliliters before applying the laccase for extended periods of time, i.e., 30 min in the Arends publication.[7] In our case, however, full saturation (~450 % air sat.) was achieved within only two minutes. This is an important realization for the time-saving performing of reactions.

After full saturation, the oxygen balloon was removed. This caused no significant change to the oxygen saturation in the reaction mixture (without laccase). Upon the addition of the laccase, the oxygen saturation in the reaction mixture decreases almost linearly from around 450% to near 0% air sat. If another oxygen balloon was applied, the re-saturation occurred.

A.7. Cyclic Voltammogramms

A.7.1. AZADOL (3)

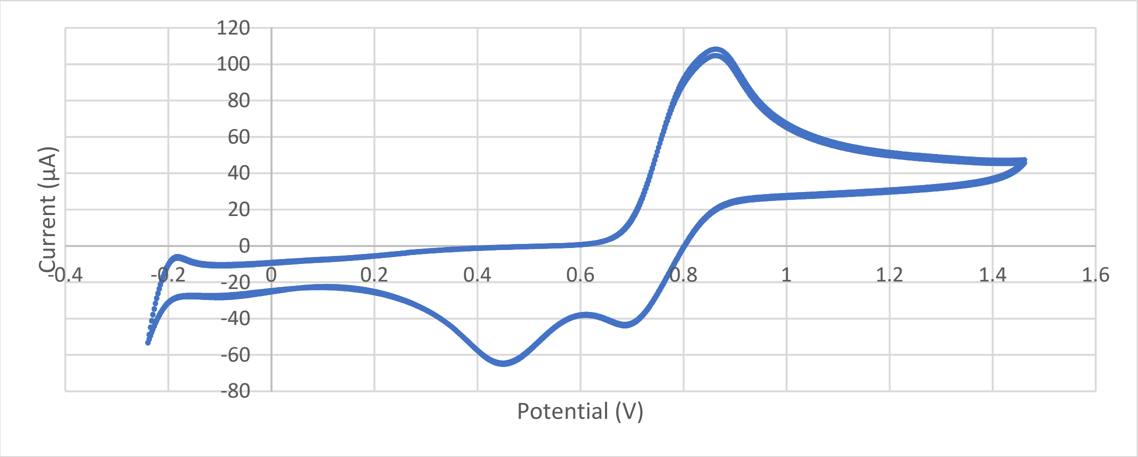

Figure 3: Cyclic voltammogram of the second and third scan of 3 scans of AZADOL (10 mM), measured in 0.1 M NaOAc-buffer, pH4.5, at a scan rate of 100 mV s^{-1}.

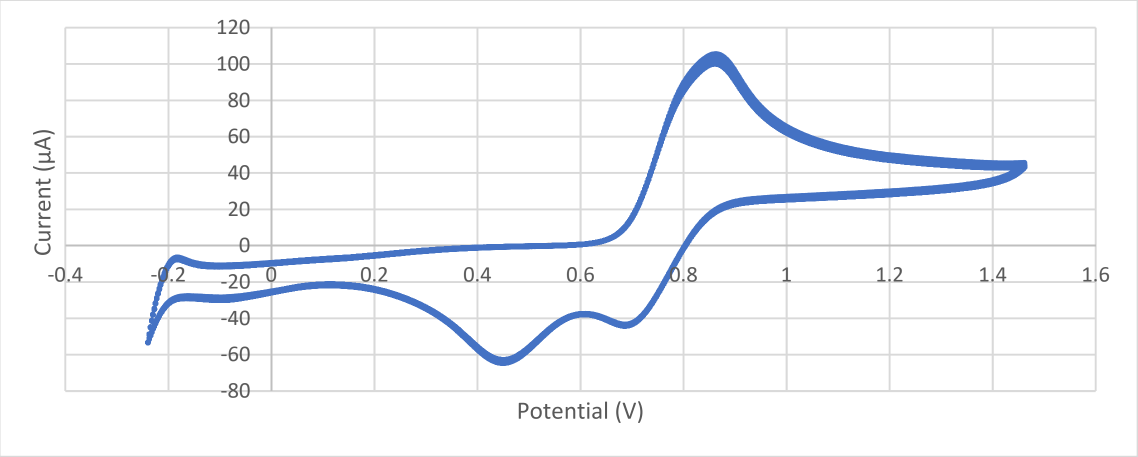

Figure 4: Cyclic voltammogram of the scans 2-100 of 100 scans of AZADOL (10 mM), measured in 0.1 M NaOAc-buffer, pH4.5, at a scan rate of 100 mV s^{-1}.

A.7.2. TEMPO (4)

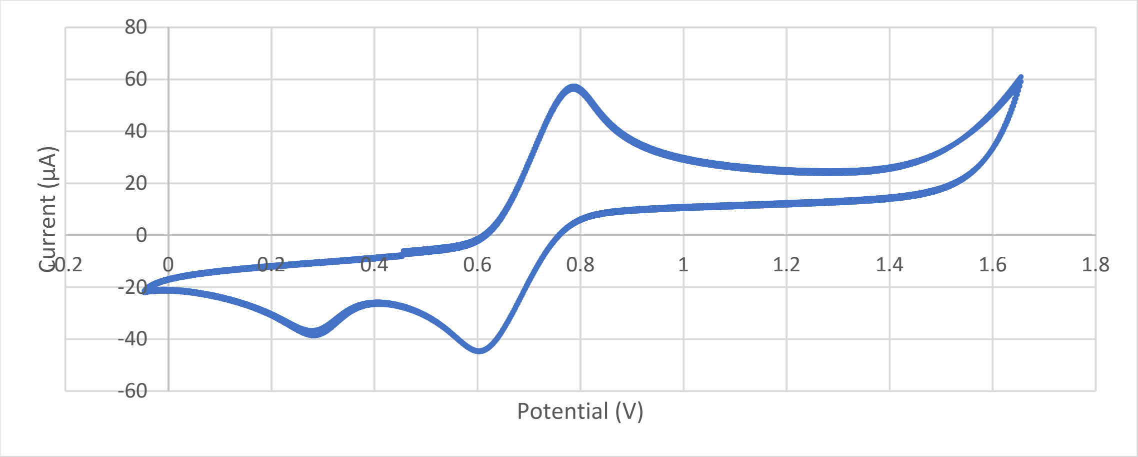

Figure 5: Cyclic voltammogram of the second and third scan of 3 scans of TEMPO (10 mM), measured in 0.1 M NaOAc-buffer, pH4.5, at a scan rate of 100 mV s^{-1}.

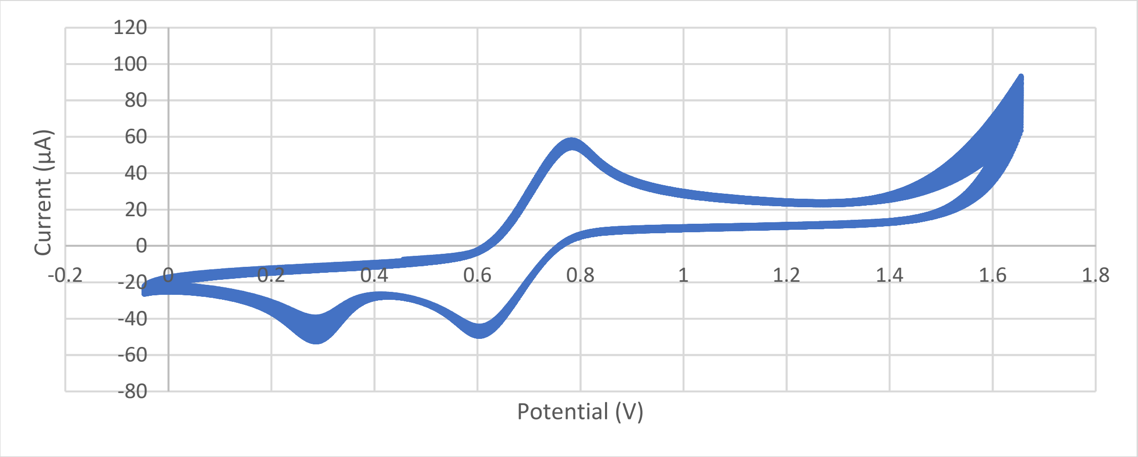

Figure 6: Cyclic voltammogram of the scans 2-100 of 100 scans of TEMPO (10 mM), measured in 0.1 M NaOAc-buffer, pH4.5, at a scan rate of 100 mV s^{-1}.
